# Development of a sensor-based site-specific N topdressing algorithm for a typical leafy vegetable

**DOI:** 10.3389/fpls.2022.951181

**Published:** 2022-08-26

**Authors:** Rongting Ji, Weiming Shi, Yuan Wang, Hailin Zhang, Ju Min

**Affiliations:** ^1^State Key Laboratory of Soil and Sustainable Agriculture, Institute of Soil Science, Chinese Academy of Sciences, Nanjing, China; ^2^Nanjing Institute of Environmental Sciences, Ministry of Ecology and Environment of the People’s Republic of China, Nanjing, China; ^3^Department of Plant and Soil Sciences, Oklahoma State University, Stillwater, OK, United States

**Keywords:** active canopy sensor, bok choy, N response index, N topdressing strategy, yield potential

## Abstract

Precise and site-specific nitrogen (N) fertilizer management of vegetables is essential to improve the N use efficiency considering temporal and spatial fertility variations among fields, while the current N fertilizer recommendation methods are proved to be time- and labor-consuming. To establish a site-specific N topdressing algorithm for bok choy (*Brassica rapa subsp. chinensis*), using a hand-held GreenSeeker canopy sensor, we conducted field experiments in the years 2014, 2017, and 2020. Two planting densities, viz, high (123,000 plants ha^–1^) in Year I and low (57,000 plants ha^–1^) in Year II, whereas, combined densities in Year III were used to evaluate the effect of five N application rates (0, 45, 109, 157, and 205 kg N ha^–1^). A robust relationship was observed between the sensor-based normalized difference vegetation index (NDVI), the ratio vegetation index (RVI), and the yield potential without topdressing (YP_0_) at the rosette stage, and 81–84% of the variability at high density and 76–79% of that at low density could be explained. By combining the densities and years, the *R*^2^ value increased to 0.90. Additionally, the rosette stage was identified as the earliest stage for reliably predicting the response index at harvest (RI_Harvest_), based on the response index derived from NDVI (RI_NDVI_) and RVI (RI_RVI_), with *R*^2^ values of 0.59–0.67 at high density and 0.53–0.65 at low density. When using the combined results, the RI_RVI_ performed 6.12% better than the RI_NDVI_, and 52% of the variability could be explained. This study demonstrates the good potential of establishing a sensor-based N topdressing algorithm for bok choy, which could contribute to the sustainable development of vegetable production.

## Introduction

Nitrogen (N) is the key nutritional driver determining crop yield and quality, especially for vegetables (Dunn et al., 2015). However, N fertilizer use is highly inefficient, and often only 10–30% of the N applied in the field can be absorbed by the crop, with a substantial amount of N loss to the environment, resulting in soil and water quality deterioration (Zhu et al., 2005; Shi et al., 2009; Song et al., 2009). The amount and timing of the N application are the two important factors determining the N-use efficiency (NUE) (Bijay-Singh et al., 2015; Zotarelli et al., 2015). In this sense, the diagnosis of field plant N status and appropriate fertilizer-application recommendations have become a critical component of ensuring high yields and good quality vegetable crops. Since small farms characterize agriculture in many countries, as reported, 73% of farms are smaller than 1 ha and 85% are smaller than 2 ha, especially in China where over 80% of households operate less than 0.6 ha of farmland (Tan et al., 2013; Lowder et al., 2016). Fine-tuned monitoring of N status of both field and crop has been challenging, and over-fertilization is a common occurrence. In these smaller-scale operations, further complicated by variable fertility histories and rotation systems, the temporal and spatial variation in soil fertility among fields has additionally restricted efficient fertilizer-N utilization (Jin and Jiang, 2002; Lowder et al., 2016). Therefore, a site-specific topdressing N-management strategy must be developed that reflects spatial variabilities and plant N availability to improve NUE in vegetable production systems (Dobermann et al., 2003).

Numerous recommendation systems have been developed and tested for improving the N-fertilizer management of vegetable crops, such as the N_min_ methods, the Kulturbegleitenden N_min_-Sollwerte System, and the N-Expert system (Burns, 2006; Schmidt et al., 2009). These existing systems are mainly based on soil testing and plant analysis; however, the cost and time required for soil and plant testing, the slow turnaround time, and high prediction errors among fields have limited the adoption of these methods by local farmers (Jin and Jiang, 2002; Ma et al., 2007). In recent years, significant progress in investigating remote-sensing technology as a real-time N diagnostic tool has been made, and related approaches have been applied to many crops (Samborski et al., 2009). Some proximal optical sensors, such as chlorophyll meters, Dualex instruments, and portable canopy sensors, have been used for determining the N status of vegetables (Gianquinto et al., 2011; Padilla et al., 2018). Among these, hand-held active canopy sensors have shown the potential to detect plant N status with high temporal and spatial resolution at the canopy level and have received much attention because of their superior operational efficiency over direct contact leaf sensors and relatively low cost compared to hyperspectral sensors (Raun et al., 2005; Xia et al., 2016).

One of the commonly used hand-held active canopy sensors is the GreenSeeker optical sensor, which measures reflectance from the plant canopy in the red and near-infrared wavelength region and provides two typical plant indices: the normalized difference vegetation index (NDVI) and the ratio vegetation index (RVI) (Yao et al., 2012). The canopy reflectance to visible light is primarily dependent on the chlorophyll content contained in the leaf palisade layer and the near-infrared reflectance depended upon the structure of the mesophyll cell and the cavities between cells, thus the NDVI and RVI indices can detect plant N status and make N recommendation (Olfs et al., 2005). Canopy characteristics have been used to guide the N management of many cereal crops, e.g., winter wheat, corn, and rice (Lukina et al., 2001; Barker and Sawyer, 2010; Ali et al., 2014). Although the GreenSeeker sensor has been widely used for cereal crops, there has been limited use for N-fertilizer management in vegetable crops due to their special nutritional characteristics, fertilization, regimes, and soil fertility level; therefore, it cannot be used by just replacing the crop in the equation, and the usability, application procedures, and accuracy of the GreenSeeker sensor need to be restudied in the new system (Tremblay et al., 2011; Ji et al., 2017). Previous studies have mainly focused on in-season N-status estimation of a specific vegetable, but protocols for determining the variable N-application rate considering the spatial variation of vegetable crops are urgently needed (Jones et al., 2007; Tremblay et al., 2011; Dunn et al., 2015; Padilla et al., 2018). Moreover, plant density is one of the most important agronomic factors in practice, and sensor readings and N requirements change at different densities (Raun et al., 2002). It is unknown whether a hand-held active canopy sensor like the GreenSeeker sensor can be used for the N management of leafy vegetables with different plant densities.

Bok choy (*Brassica rapa subsp. chinensis*) is a popular leafy vegetable that originated in China over 1,500 years ago. Currently, it is widely cultivated and consumed in China and north-eastern Asia for its antioxidant benefits and winter hardiness (Heimler et al., 2006; Zhang et al., 2014). In particular, it is a popular leafy vegetable in the Taihu Lake region of southern China, and compared with a previous study, there seems to be a large opportunity for N-fertilizer reduction for bok choy in this region (Zhang et al., 2016). Key questions, therefore, were whether the GreenSeeker sensor may be employed to produce N-fertilizer recommendations for bok choy and whether a sensor-based topdressing N-management strategy could reduce N-fertilizer applications without sacrificing yields. We hypothesize that the GreenSeeker sensor would be used to make N recommendation of bok choy with different densities, and the N fertilizer rate would be reduced considering temporal and spatial soil fertility. In our study, a 3-year field experiment of bok choy with different densities and N-application rates was conducted in the Taihu Lake region. The objectives were to (i) determine whether the sensor-based N diagnosis model could be applied to bok choy with different plant densities, (ii) identify the suitable growth stage and plant index between NDVI and RVI for bok choy to make accurate predictions of yield without additional N (YP_0_) and the response index (RI) with topdressing N, and (iii) develop an accurate sensor-based N-fertilizer topdressing strategy for bok choy to improve the N-use efficiency.

## Materials and methods

### Study site

**FIGURE 1:**
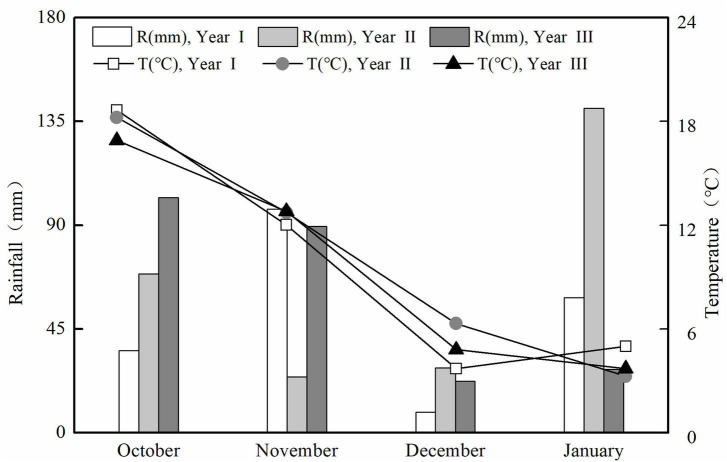
Monthly precipitation (R, mm) and average air temperature (*T*, °C) at the experiment site in cropping Years I, II, and III.

Field experiments with bok choy (*Brassica rapa subsp. chinensis*) were conducted in 2014 (Year I), 2017 (Year II), and 2020 (Year III) in Yixing (31°16′N, 119°54′E), Jiangsu Province, which is located in the center of the Taihu Lake region in south-eastern China. This region has a sub-tropical monsoon climate, with a mean annual air temperature and rainfall of 15.7°C and 1,177 mm, respectively. The distribution of precipitation and temperature during the experimental period is shown in Figure 1. Bok choy was planted in a new vegetable field (a traditional bok choy-radish-cabbage rotation was applied in each field every year), and the soil type at the experiment site was classified as hydroagric Stagnic Anthrosol by the Chinese soil taxonomic classification (Gong, 1999), with a pH (H_2_O) of 6.25, an electrical conductivity (EC) of 0.48 mS cm^–1^, and the soil organic matter content, total N, NO_3_^–^-N, and NH_4_^+^-N were 18.5 g kg^–1^, 1.06 g kg^–1^, 84.2 mg kg^–1^, and 24.6 mg kg^–1^, respectively.

### Experimental design

In Years I and II, 2,680 kg ha^–1^ manure (consisting of 45 kg N ha^–1^) and 24 kg P ha^–1^ from calcium superphosphate and 47 kg K ha^–1^ using potassium sulfate were applied to all treatments at transplanting as basal fertilizer. In addition, four chemical N rates were employed, designated as N1, N2, N3, and N4 (0, 64, 112, and 160 kg N ha^–1^, respectively), supplied as urea. The inorganic N fertilizer was applied in two splits: 30% at transplanting and 70% at the beginning of the 5–6 true-leaf stage. A randomized complete block design with three replications was used, and each plot measured 33.6 m^2^ (7.0 m × 4.8 m). To test the influence of plant densities, a high density (HD) was employed in Year I, with 123,000 plants ha^–1^, while in Year II, a low density (LD) with 57,000 plants ha^–1^ was used. In Year III, a split experiment was conducted with two densities of HD and LD, and each plot measured 16.8 m^2^ (3.5 m × 4.8 m). And two no-N fertilizer treatments (only 24 kg P ha^–1^ from calcium superphosphate and 47 kg K ha^–1^ using potassium sulfate) under HD and LD, with three replications each, were added to test the effect of soil background nitrogen supply potential.

Seedlings were grown in a small nursery first and then transplanted at the two-leaf stages into the experimental plots. In Year III, 24 plots (4.0 m^2^ plot each^–1^) located adjacent to the experiment site were established as a validation field; a moderate plant density (87,500 plants ha^–1^) was chosen in the validation field. The N management of the validation field was designed according to local farmer practice, and the plots were arranged randomly. The corrective N-management strategy was tested to determine N fertilizer application at the rosette stage using the experimental data, and the detailed experimental design is shown in Table 1.

**TABLE 1:** Detailed experimental design.

Year	Plant density	Transplanting date	Harvest date	Nitrogen application rate (kg N ha^–^^1^)	Plot area (m^2^)	Experimental function
Year I	123,000 plants ha^–1^	22 October	20 December	N1 (45+0), N2 (45+64), N3 (45+112), N4 (45+160)	33.6	Modeling
Year II	57,000 plants ha^–1^	30 October	7 January	N1 (45+0), N2 (45+64), N3 (45+112), N4 (45+160)	33.6	Modeling
Year III	123,000 and 57,000 plants ha^–1^	3 November	13 January	N0 (0), N1 (45+0), N2 (45+64), N3 (45+112), N4 (45+160)	16.8	Modeling
Year III	87,500 plants ha^–1^	Farmer practice	Farmer practice	Farmer practice	4.0	Validation

### Data collection

A GreenSeeker™ (Trimble Inc., Sunnyvale, CA, United States) hand-held sensor was used to collect reflectance data using red (671 ± 6 nm) and near-infrared (780 ± 6 nm) radiation. The sensor was positioned horizontally and parallel to the crop row approximately 60 cm above the crop canopy in each plot except the border rows by holding the GreenSeeker and walking at a constant speed, and the average of four measurements of independent rows from each plot was reported. Two vegetation indices (NDVI and RVI) were calculated by the internal software, and the calculations are as follows (Tremblay et al., 2009):


(1) $$NDVI=\frac{\rho_{NIR}-\rho_{R}{}_{ed}}{\rho_{NIR}+\rho_{R}{}_{ed}}$$

(2) $$RVI=\frac{\rho_{NIR}}{\rho_{R}{}_{ed}}$$

Where:

*ρ _*NIR*_* = reflectance at the near-infrared (NIR) region;

*ρ _*Red*_* = reflectance at the red region.

Sensor readings were collected at four growth stages (the 5–6 true-leaf, rosette, cupping, and harvest stage), and the exact sensing dates are presented in Table 2. The yield of each plot was determined from the aboveground fresh biomass of the bok choy adjusted to the water content of 90%. As for the validation field, the sensor readings of four stages and harvest yield were collected like those in the experimental plots. Additionally, the average N concentration of harvest bok choy was estimated by randomly sampling plants from 24 validation fields in the research region. Plant samples were oven-dried at 105°C for 30 min, then dried at 70°C to a constant weight, and later ground into fine powder to determine N concentration by a modified Kjeldahl digestion method (Xia et al., 2016).

**TABLE 2:** Sensing dates of bok choy at the four specific growth stages.

Year	5–6 true-leaf stage	Rosette stage	Cupping stage	Harvest stage
Year I	November 10	November 22	December 2	December 15
Year II	November 15	November 27	December 14	December 28
Year III	November 17	December 2	December 20	January 11

### YP_0_, YP_N_, RI calculation, and N-fertilizer use efficiency evaluation

To evaluate the potential of using the GreenSeeker sensor to estimate bok choy yield potential without additional N (YP_0_), only treatments that received preplant N applications were used for the yield prediction model (including N1 treatment in Years I and II and N0 and N1 treatment in Year III). And YP_0_ can be estimated by the empirical exponential relationship between yield and NDVI or RVI measurements of all N treatments collected by the GreenSeeker sensor, YP_0_ = a*e^b*NDVI^ or YP_0_ = a*e^b*RVI^ (Teal et al., 2006; Ji et al., 2017). The YP_N_ was calculated by multiplying the YP and RI_Harvest_ values estimated by RI_NDVI_ (RI_RVI_) (Raun et al., 2005; Yao et al., 2012). Johnson and Raun (2003) introduced the response index (RI) as a measure of a plant’s response to additional N fertilizer. The RI is determined by comparing treatments or farm practice with a reference plot, which was traditionally used as the highest N-rate plot and represents an area where N is not a yield-limiting factor (Johnson and Raun, 2003; Lofton et al., 2012a). In our study, a 205 kg N ha^–1^ (160 kg N ha^–1^ from chemical fertilizer plus 45 kg N ha^–1^ from manure) treatment for bok choy was used as the reference plot. RI_NDVI_ (RI_RVI_) was calculated by dividing the mean NDVI (RVI) of the reference plots by the average measurement of other N-treated plots, while RI_Harvest_ was calculated by dividing the mean yield of the reference plots by the yield of other N-treated plots.

The recovery efficiency (RE) and agronomic efficiency (AE) were computed as follows to assess the N-use efficiency of N-fertilizer input (Ali et al., 2014):


(3) $$\mathrm{RE}\left(\%\right)=\frac{\begin{array}{c} \mathrm{total}N\mathrm{uptake}\mathrm{in}N\mathrm{fertilized}\mathrm{plot}-\mathrm{total}N\mathrm{in}\mathrm{no}N\mathrm{plot} \end{array}}{\begin{array}{c} \mathrm{quantity}\mathrm{of}N\mathrm{fertilizer}\mathrm{applied}\mathrm{in}N\mathrm{fertilized}\mathrm{plot} \end{array}}\times 100\%$$

(4) $$\mathrm{AE}\mathrm{kg}\mathrm{yield}/\mathrm{kg}N\mathrm{applied}=\frac{\mathrm{yield}\mathrm{in}N\mathrm{fertilized}\mathrm{plot}-\mathrm{yield}\mathrm{in}\mathrm{no}N\mathrm{plot}}{\mathrm{quantity}\mathrm{of}N\mathrm{fertilizer}\mathrm{applied}\mathrm{in}N\mathrm{fertilized}\mathrm{plot}}$$

### Statistical analysis

NDVI, RVI, and yield variation between different N fertilizer treatments and plant densities were subjected to one-way and two-way analyses of variance (ANOVA) and a Duncan multiple-comparison test with SPSS (ver. 20.0 for Windows, SPSS Inc., Chicago, IL, United States). All the regression coefficients in the study were calculated and plotted by Origin 8.5 (OriginLab Corporation, Northampton, MA, United States).

## Results

### Yield responses to different N rates and plant densities

**FIGURE 2:**
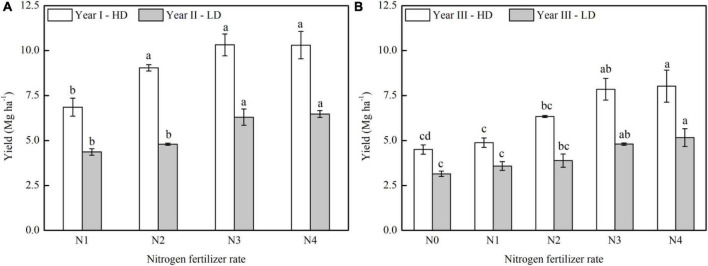
Yield responses of bok choy to different chemical N fertilizer rates in Years I, II **(A)**, and Year III **(B)**. Treatments N0, N1, N2, N3, and N4 received 0, 45, 109, 157, and 205 kg⋅N⋅ha^–1^ in the growth season, respectively. Different letters indicate significant differences among various N application rates in the specific year at the *P* < 0.05 level.

Bok choy yields showed a significant response to different N rates and densities (Figure 2; Supplementary Table 1). The yields were greatly enhanced by increasing N rates in both years and densities. The yields of bok choy under N2, N3, and N4 treatments were increased by 31.9, 50.6, and 50.4% in Year I and 9.8, 44.4, and 48.4% in Year II. In Year III, yields under N1 to N4 treatments were 8.5–78.4% and 13.8–63.9% higher than the N0 treatment, and 29.8–64.3% and 7.9–40.9% higher than the N1 treatment at high- or low density, respectively. The yields increased as the N rate increased up to N3, but no further significant increase was observed beyond N3, suggesting that 112 kg N ha^–1^ as urea accompanied 45 kg N ha^–1^ from organic fertilizer was the optimum N rate for bok choy. As shown in Figure 2A, the yield difference between Years I and II was rather significant, and the actual yield of bok choy in Year II was 36.3–46.9% lower than that in Year I, suggesting that plant density has a significant influence on the final yields of bok choy at harvest, since the plant population in Year II was less than half of that in Year I. The yield in Year III also confirmed the effect of plant density, and 26.6–40.2% gap was observed (Figure 2B). Also, because of frost damage during the growing period, the yield in Year III was significantly lower than that in Years I and II, but a similar yield response trend was observed. Thus, the yield of bok choy was significantly increased as the N-application rate up to 157 kg N ha^–1^, and plant densities may have a remarkable influence on yields at harvest.

### In-season prediction of YP_0_

Current N recommendation strategies are mainly “yield-based,” and YP_0_ is the possible attainable yield with no additional N (Raun et al., 2002). In practice, accurately estimating YP_0_ at the early stage was the first step in determining a site-specific N-management strategy to achieve optimum yields. Compared with three common types of regression equations (Supplementary Table 2), the empirical exponential model was used to determine the relationship between YP_0_ and the sensor-based vegetation indices (NDVI and RVI) for bok choy across growth stages (Table 3). NDVI and RVI could accurately estimate YP_0_, but the relationship was not robust across all stages. At the 5–6 true-leaf stage, a low correlation was noted between sensor measurements and YP_0_, and the determination coefficient ranged from 0.68 to 0.72 (Table 3). The highest *R*^2^ was obtained at the rosette stage, and 81–84% of YP_0_ of the variability under HD and 76–79% of the variability under LD could be explained by RVI and NDVI. However, at the cupping and harvest stage, a significantly weaker relationship was observed, and 1–21% of the yield variations could be explained (Table 3). In a comparison between the two sensor-based vegetation indices, NDVI and RVI, almost the same accuracy in predicting YP_0_ was found at each stage.

**TABLE 3:** Relationships between sensor-based measurements (NDVI and RVI) and yield potential without additional topdressing N application (YP_0_) of bok choy at different densities of high density (HD) and low density (LD).

Growth stage	Index	HD			LD		
		Equation	*R* ^2^	RMSE^a^	Equation	*R* ^2^	RMSE
5–6 True-Leaf	NDVI	*y* = 3.05e^3.92x^	0.68	0.73	*y* = 2.60e^1.65x^	0.70	0.35
	RVI	*y* = 1.90e^0.63x^	0.68	0.74	*y* = 1.75e^0.48x^	0.72	0.34
Rosette	NDVI	*y* = 1.13e^4.01x^	0.81	0.57	*y* = 1.20e^3.75x^	0.79	0.31
	RVI	*y* = 1.05e^0.72x^	0.84	0.53	*y* = 0.76e^0.86x^	0.76	0.32
Cupping	NDVI	*y* = 1.08e^3.04x^	0.16	1.20	*y* = 5.91e^–1.20x^	0.03	0.64
	RVI	*y* = 1.85e^0.33x^	0.15	1.20	*y* = 6.09e^–0.22x^	0.03	0.64
Mature	NDVI	*y* = 4.98e^0.15x^	0.01	1.30	*y* = 0.99e^2.88x^	0.20	0.58
	RVI	*y* = 4.64e^0.044x^	0.01	1.31	*y* = 1.16e^0.43x^	0.21	0.58
Pooled	NDVI	*y* = 11.76 x−0.062DAT+2.72	0.42	0.96	*y* = 8.23x−0.053DAT+3.07	0.36	0.65
	RVI	*y* = 1.83x−0.059DAT+2.80	0.40	0.98	*y* = 1.43x−0.044DAT+2.45	0.35	0.65

^a^RMSE means the root mean square error; DAT means days after transplanting.

**FIGURE 3:**
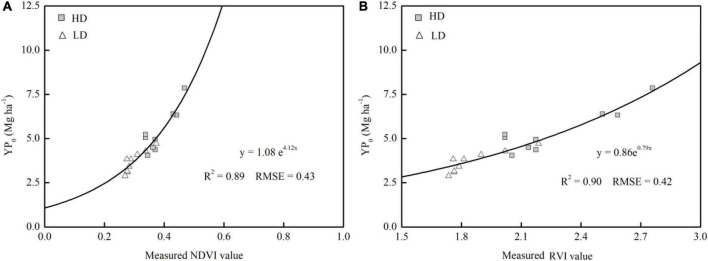
Relationship between in-season estimates of yield potential without additional N topdressing (YP_0_) at the rosette stage calculated using the normalized difference vegetation index (NDVI) **(A)** and the ratio vegetation index (RVI) **(B)** in Years I, II, and III.

Regression analyses (Table 3) suggest that the rosette stage was the most appropriate stage for YP_0_ prediction in both years and densities, which coincides with the optimum time for topdressing N to bok choy during production. However, for practical application purposes, it would be more convenient to build a general model to predict the yield potential of vegetables with different plant densities. The fitting curves of pooled sensor readings (NDVI and RVI) and YP_0_ with different densities among years at the rosette stage are presented in Figure 3. By combining all years-densities data, the sensor-based yield prediction model could explain 89–90% of the YP_0_ variation. Between the two indices, RVI performed similarly to NDVI. Additionally, the plant density showed a significant effect on YP_0_, and the YP_0_ under HD treatments were much higher than that of LD; thus, the data points for HD treatment are located on the upper section of the line, while LD treatment data are positioned on the lower portion of the fitting curves; however, the trend was similar for both densities (Figure 3).

### In-season prediction of N response index

**FIGURE 4:**
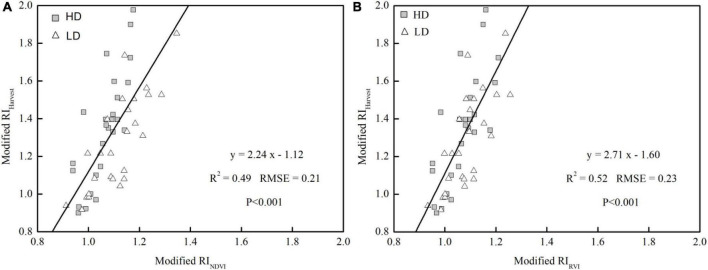
Relationships between the response index calculated with yield (RI_Harvest_) and the response index calculated with NDVI (RI_NDVI_) **(A)** and RVI (RI_RVI_) **(B)** at the rosette stage of bok choy growth in Years I, II, and III.

Aside from the accurate prediction of YP_0_, the degree to which a crop responded to additional N fertilizer was also a key component in determining the appropriate N-management strategy. Table 4 shows the relationships between RI_Harvest_ and RI_NDVI_ (or RI_RVI_), which were computed from the NDVI (or RVI) readings collected at each stage. RI_NDVI_ and RI_RVI_ were significantly correlated with RI_Harvest_, but the parameters were different among the stages. The sensor-based RIs can accurately predict RI_Harvest_ after the 5–6 true-leaf stage, and the rosette stage was the earliest stage for which RI_Harvest_ estimation was conducted, with *R*^2^ values of 0.59–0.67 under HD treatment and 0.53–0.65 under LD treatment. After the rosette stage, a weaker relationship was observed for both RI_NDVI_ and RI_RVI_; furthermore, those later stages were too late for topdressing N. Therefore, the rosette stage was the most appropriate stage for conducting the RI_Harvest_ prediction, which was consistent with the growth stage for YP_0_ prediction. After using a combined equation to explain the variation across years and densities at this stage, a weaker, but still significant, relationship was observed relative to that of individual densities. The accuracy of the RVI-based model and the RMSE value was 6.12% higher and 8.70% lower, respectively, than that seen with the NDVI-based model (Figure 4), with RVI explaining 52% of RI_Harvest_ variability. This result confirms the superiority of the RVI-based model in explaining RI_Harvest_ variability.

**TABLE 4:** Relationship between RI_NDVI_ and RI_RVI_ with RI_Harvest_ (*y* in the equations) of bok choy across years at different densities of high density (HD) and low density (LD).

Growth stage	Index	HD			LD		
		Equation	*R* ^2^	RMSE	Equation	*R* ^2^	RMSE
5–6 True-Leaf	RI_NDVI_	*y* = 1.73x−0.45	0.31	0.27	*y* = 1.13x+0.10	0.26	0.23
	RI_RVI_	*y* = 1.88x−0.59	0.17	0.30	*y* = 1.58x−0.35	0.16	0.24
Rosette	RI_NDVI_	*y* = 3.57x−2.44	0.67	0.19	*y* = 1.79x−0.73	0.65	0.16
	RI_RVI_	*y* = 3.18x−2.05	0.59	0.21	*y* = 1.87x−0.76	0.53	0.18
Cupping	RI_NDVI_	*y* = 3.11x−1.95	0.60	0.21	*y* = 1.65x−0.59	0.57	0.17
	RI_RVI_	*y* = 1.80x−0.64	0.62	0.20	*y* = 1.47x−0.40	0.58	0.17
Mature	RI_NDVI_	*y* = 3.20x−1.95	0.44	0.24	*y* = 2.01x−0.87	0.52	0.18
	RI_RVI_	*y* = 0.51x−0.62	0.59	0.21	*y* = 1.44x−0.29	0.49	0.19
Pooled	RI_NDVI_	*y* = 2.71x+0.0019DAT-1.66	0.31	0.21	*y* = 1.34x−0.0015DAT-0.19	0.41	0.18
	RI_RVI_	*y* = −0.57x+0.0067DAT+1.68	0.20	0.27	*y* = −1.34x+0.0017DAT+2.60	0.41	0.18

### Model validation

**FIGURE 5:**
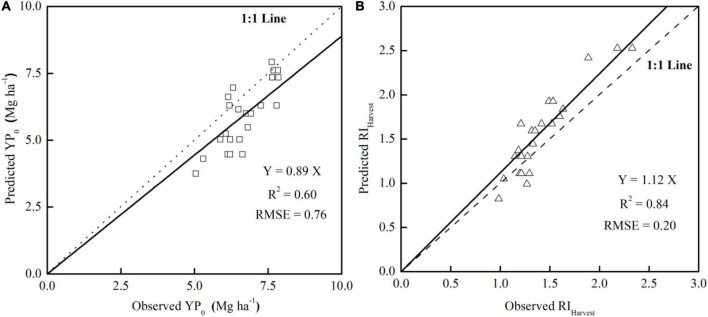
Relationship between the observed and predicted YP_0_ **(A)** and RI_Harvest_ **(B)** in the validation field.

The exponential and linear equations developed from the RVI measurements at the rosette stage were used to predict YP_0_ (YP_0_ = 0.86e^0.79 × RVI^, *R*^2^ = 0.90) and RI_Harvest_ (RI = 2.71 × RI_RVI_-1.60, *R*^2^ = 0.52) at either the high or low plant density of bok choy. To validate the reliability of the YP_0_ and RI_Harvest_ prediction model for different densities, we tested the model using an independent data set obtained from the validation field with a moderate plant density (87,500 plants ha^–1^) in Year III. According to the results of the YP_0_ and RI prediction models, the rosette stage was the most suitable growth stage, so RVI measurements obtained at that stage were used for validation to determine the relationship between the predicted YP_0_ and RI_Harvest_ and the actual values. As shown in Figure 5, the observed YP_0_ was highly correlated with the predicted YP_0_ (*R*^2^ = 0.60) in almost a 1:1 relationship, with a slope of 0.89. For RI_Harvest_, the predicted value was slightly higher than the observed results, but a good relationship (*R*^2^ = 0.84) was observed, with a slope of 1.12. In general, the data points of the two validation equations were all scattered evenly around the 1:1 line. This confirms the potential of applying the in-season YP_0_ and RI_Harvest_ prediction model to various plant populations.

### Strategy for in-season site-specific N management for bok choy

In practice, the potential yield with added N fertilization (YP_N_) can be estimated by multiplying YP_0_ and RI; the N requirement is then determined by multiplying the yield response by plant N concentration and average NUE (Raun et al., 2005). In our study, the RVI is superior to the NDVI measurement for predicting YP_0_ and RI_Harvest_.

Thus, the site-specific N-topdressing algorithm for bok choy is expressed as follows (Raun et al., 2005; Yao et al., 2012):


(5) $$N_{rate}=\frac{[(YP_{0}\times RI)-YP_{0}]\times N\%}{NUE}$$

where YP_0_ = 0.86e^0.79 × RVI^ and RI = 2.71 × RI_RVI_-1.60.

The NUE used in the formula is defined as the NUE of the additional N input. However, there is no consensus yet on determining the vegetables’ NUE, as it depends on various soil and environmental factors, fertilizer management, and treatments applied (Barker and Sawyer, 2010; Li et al., 2017). In our study, the average NUE in the research region of vegetable crops topdressing N fertilizer was approximately 15%; thus, the NUE in the formula was initially set at 15% for bok choy. Additionally, the N% expressed in the model was the average N content of bok choy at harvest; according to our investigation, the harvested N concentration of bok choy in the Taihu Lake region was approximately 5.5% (Supplementary Table 3). Based on the above discussion, the NFOA for bok choy can be established.

### Evaluation of the sensor-based N topdressing algorithm of bok choy

Practically, a field optimum N rate is typically recommended for the whole region and then adjusted to the specific rate for a certain field based on the spatial variation (Zhu, 2006; Yao et al., 2012). Therefore, a practical precision-driven N topdressing algorithm for bok choy was proposed for the Taihu Lake region, using 45 kg N ha^–1^ from manure as basal N fertilizer plus a 112 kg N ha^–1^ chemical N fertilizer split-applied at the transplanting and 5–6 true-leaf stage, at 30 and 40% proportion according to the nutrient requirement pattern followed by determination of the need for additional topdressing N at the rosette stage using the RVI-based NFOA (Zanão Júnior et al., 2005).

To address whether the sensor-based topdressing N-management strategy could reduce N fertilizer application without sacrificing yields, we examined the proposed N-management strategy using the plots with approximately equal N application rates in Years I, II, and III at high or low density. The data in Table 5 show the outcomes of normal farm practice and the sensor-based N-topdressing strategy. A similar yield was obtained under the traditional farm fertilizer practice and the sensor-based topdressing strategy, at both high and low density (Table 5). According to our calculation, the amount of N recommended ranged from 43.41 to 58.77 kg N ha^–1^ under the high-density treatment and from 29.07 to 37.97 kg N ha^–1^ under the low-density treatment. The recommended rate was significantly lower than that employed as part of the normal farm practice, especially for the low-density treatment. When appropriately prescriptive N fertilizer amounts were applied and a sensor-based corrective N strategy was conducted, average increases of 14.91 and 31.17% of RE and 3.60 and 4.58 kg yield/kg N of AE by fertilizer N over normal farm practice were observed at high and low densities, respectively. Therefore, a reduction of 18.16–32.65% N can be achieved by using the sensor-based approach compared to N-management practice in conventional farming. Additionally, when compared with the optimum N rate at 157 kg N ha^–1^, the amount of the sensor-based NFOA was almost equal to, or slightly less than, the optimum N rate, and the fertilizer rate gap between the NFOA-calculated fertilizer rate and the optimum N rate reflecting the soil fertility variation among plots.

**TABLE 5:** Estimation of sensor-based topdressing N amounts at different densities using the experimental data for bok choy.

Plot	Fertilizer N application (kg N ha^–1^)			YP_0_ (Mg ha^–1^)	Yield/YP_N_ (Mg ha^–1^)	RE (%)	AE (yield/kg N)	Reduced nitrogen fertilizer (kg N ha^–1^)
	Fixed N rate ^a^	Recommended N rate ^b^	Total					
H^c^-Plot 1	109	58.77	167.77	8.33	10.74	19.49	23.18	37.23
H-Plot 2	109	54.41	163.41	8.74	11.10	20.84	25.54	41.59
H-Plot 3	109	56.99	165.99	7.86	10.19	17.88	20.12	39.01
H-Plot 4	109	48.88	157.88	5.42	7.33	9.85	17.90	47.12
H-Plot 5	109	43.41	152.41	5.48	7.17	9.64	17.54	52.59
H-Plot 6	109	47.45	156.45	5.31	7.15	9.33	16.97	48.55
L^d^-Plot 7	109	37.97	146.98	4.83	6.38	12.45	13.74	58.02
L-Plot 8	109	37.87	146.88	5.19	6.74	13.81	16.20	58.12
L-Plot 9	109	36.08	145.08	5.19	6.67	13.71	15.92	59.92
L-Plot 10	109	31.73	140.73	3.88	5.12	7.69	13.99	64.27
L-Plot 11	109	34.44	143.44	4.00	5.34	8.40	15.27	61.56
L-Plot 12	109	29.07	138.07	3.76	4.89	6.93	12.60	66.93

^a^Fixed N rate suggested the N application rate of 45 kg N ha^–1^ organic fertilizer and 19 kg N ha^–1^ chemical N fertilizer at transplanting as base fertilizer plus 45 kg N ha^–1^ chemical N fertilizer at 5–6 true-leaf stage according to the nutrient requirement pattern. ^b^Recommended N rate suggested the topdressing N application rate calculated at the rosette stage by the site-specific N-topdressing algorithm. ^c^H, high-density plot; ^d^L, low-density plot. RE and AE stand for N recovery efficiency and agronomic efficiency.

## Discussion

### Sensor-based approaches used for assessing the crop N management of vegetable crops

Optical sensors have been a promising alternative approach for non-destructive crop monitoring (Ali et al., 2020). However, few studies have paid attention to the sensor-based N fertilizer management in vegetable production systems. This phenomenon was partly owing to the complicated canopy architecture of vegetable crops which would complicate the work of the sensor (Padilla et al., 2018). The RVI determined by the GreenSeeker sensor had a significant relationship with YP_0_ (*R*^2^ = 0.90) and RI_Harvest_ (*R*^2^ = 0.52) (Figures 3, 4), the RVI performed better than the NDVI on account of its higher sensitivity *vis-a-vis* the former during the early vegetation and maturity stages, especially at high plant densities (Li et al., 2013). Given the different fitting results of the YP_0_ and RI_Harvest_ prediction model in Years I, II, and III, it may be influenced by the climate differences between years to some degree (Figure 1), and this need to have more validation (Raun et al., 2005; Ji et al., 2017). When considering the variation in yield between years, which may be due to the impact of vegetable stubble changes, pests and diseases pests during a particular year, and adverse weather, it did not influence the use of optical sensors (Liu et al., 2008; Li et al., 2017). Moreover, the YP_0_ and RI_Harvest_ prediction model would not be affected even if the relatively yield fluctuates (Figures 3, 4). To sum up, compared with the results for rice and sugarcane, the findings of our study were as good as, or better than, the predictions for other crops (Lofton et al., 2012b; Yao et al., 2012); therefore, the YP_0_ and RI index of bok choy could be reliably assessed by using the GreenSeeker sensor.

### A sensor-based site-specific N topdressing strategy of bok choy

Determining the correct amount of topdressing N is a critical step toward enhancing NUE and ensuring the productivity and quality of vegetable crops (Zhou et al., 2017). However, the complicated canopy architecture and the relatively short growth cycle of bok choy and other vegetables relative to that of grain crops (e.g., corn and rice) has caused difficulty in determining the proper timing and amounts of topdressing N for vegetables (Lukina et al., 2001; Barker and Sawyer, 2010; Lofton et al., 2012b; Ali et al., 2014; Xia et al., 2016). In addition, the coexistence model of chemical N fertilizer and organic fertilizer in the vegetable production system and the uncertainty of the N mineralization capacity of organic fertilizer significantly increases the difficulty of determining the amount of topdressing N (Chen et al., 2006; Ren et al., 2014). As found, the total yield of N4 in Years I, II, and III was slightly lower or equal to that of N3 treatment, and no significant difference was observed, therefore the N4 rate was selected as the non-N limiting treatment. And an average organic fertilizer application rate used by local farmers (about 45 kg N ha^–1^) from manure was applied as the basal fertilizer to all treatments as part of the total N input, and, thus, the amount of topdressing N could be quantitatively assessed and the sensor-based NFOA was used in our study to establish the amount of topdressing N fertilizer at the rosette stage of bok choy Eq. 5, and the recommended N-fertilizer strategy was thus proposed for the Taihu Lake region (Zanão Júnior et al., 2005; Zhu, 2006; Yao et al., 2012). According to preliminary estimation, the sensor-based topdressing strategy could reduce the total N input by 18.16–32.65% without sacrificing yields and could improve RE by 14.91–31.17% and AE by 16.10–30.82% over traditional farming practices (Table 5), which is consistent with findings for rice, where site-specific sensor-based N management increased the partial factor productivity of farmers by 48% without significantly affecting grain yield (Li et al., 2009).

### Effect of plant density on yield and the sensor-based prediction model

Plant density is one of the most important agro nomic management practices that influence crop yields (Lama et al., 2018; Adams et al., 2019). The yield of high-density plots (123,000 plants ha^–1^) was 26.6–46.9% higher than that of low-density plots (57,000 plants ha^–1^) under each N-application rate (Figure 2). This result is consistent with the finding for willow, where a plant density of 20,000 plants ha^–1^ resulted in a higher yield than a density of 15,000 plants ha^–1^ (Wilkinson et al., 2007). Plant density can also influence vegetation coverage and measured vegetation indices and, thus, affects sensor-based N predictions (Yao et al., 2015). In addition, as the plant population or density increases, vegetation coverage also increases, and the by-plot coefficient of variation decreases (Supplementary Table 4; Arnall et al., 2006), thus the effect of plant density on the YP_0_ and RI_Harvest_ predictions was assessed. The combined year-density fitting curves showed a more precise prediction of YP_0_ than the individual ones, with the *R*^2^ value increasing by 5.62–15.56% (Table 3 and Figure 3), suggesting that plant density would affect sensor readings, and higher plant densities resulted in higher sensor values; however, plant density had no effect on YP_0_ predictions. The validation result of the YP_0_ and RI_Harvest_ predictions, at a moderate plant density (87,500 plants ha^–1^), confirmed the possibility of applying the estimation model to various densities (Figure 5), which is consistent with previous research on rice (Xue et al., 2014).

### Optimum timing for making accurate YP_0_ and RI_Harvest_ predictions

The growth stage is an important factor in predicting YP_0_ and RI_Harvest_ by canopy sensors (Yao et al., 2012; Bijay-Singh et al., 2015). The NDVI and RVI had a significant relationship with YP_0_, but the relationship was not stable across different stages (Table 3), which was similar to that for rice, where NDVI and RVI could explain at most 50% of the aboveground biomass variability due to interference from soil and water background in the early growth stages (Gnyp et al., 2014). The totally poor accuracy of the YP_0_ prediction model observed under low density relative to that observed at high density was partly due to the relatively lower plant density and stronger soil background interference in the growth stages (Table 3; Cao et al., 2015). At the rosette stage, the accuracy of the YP_0_ prediction increased for both years and densities, and the *R*^2^ was significantly higher than that in other growth stages. However, at later stages, the relationship seemed weak, as the plant canopy began to close and the sensor became saturated at this stage and could not make satisfactory predictions (Ali et al., 2014; Ji et al., 2017). Thus, the rosette stage was the most appropriate stage for YP_0_ estimation.

Similarly, the RI_Harvest_ prediction was variable across stages (Table 4). For both the NDVI- and RVI-based RI_Harvest_ prediction models, accuracy was relatively low at the 5–6 true-leaf stage for the possible effect of input N had not yet manifested. At later growth stages, the *R*^2^ of the RI_Harvest_ prediction model increased by over 90.32–150.00% that of the 5–6 true-leaf stage in experimental years. However, sensor measurements at the harvest stage were not conducted, since it is not desirable to apply N at this stage of growth (Xue et al., 2014). That is consistent with the results of Lofton et al. (2012b) for sugarcane, where a short interval after fertilization was not suitable for estimating the RI_Harvest_ of cane tonnage and sugar yield, but the RI_NDVI_ at 4 and 5 weeks after fertilization could capture changes in RI_Harvest_ (Lofton et al., 2012b). Therefore, the rosette stage represented the optimum stage for conducting RI_Harvest_ prediction, and this period was also ideal for topdressing N if needed.

## Conclusion

The development of a sensor-based N-management strategy can provide an effective tool for site-specific topdressing recommendations. It was hypothesized that the GreenSeeker sensor could be used for *in situ* N fertilizer management and topdressing recommendation of bok choy. Through the three-year field experiment, it was confirmed that the active canopy sensor could be used to reliably estimate the YP_0_ and RI_Harvest_ of bok choy at the rosette stage, and the RVI functioned better than the NDVI. In addition, the across-years/densities models and the validation results confirmed the potential of the sensor-based model to be applied to different plant densities. A practical and environmentally friendly N management strategy for bok choy in the Taihu Lake region is proposed, and it consists of using 45 kg N ha^–1^ from manure as basal N fertilizer and a 112 kg N ha^–1^ split-applied at the transplanting and 5–6 true-leaf stage, at 30 and 40% of the total. The remaining N should be based on the optical sensor prediction at the rosette stage. According to our estimation, a reduction of 1/5–1/3 N can be achieved by using the sensor-based approach. This strategy can improve the NUE of bok choy, is more suitable for practical applications, and has the potential to contribute to the sustainable development of vegetable crops. The current experiment represents an important field validation for the development and implementation of vegetable N-management strategies but was conducted with a limited site-year design, and NFOA parameters warrant optimization and testing with more data in future work. Further work is needed to validate the fertilizer reduction potential in on-farm applications and to explore its applicability with more site-year data.

## Data availability statement

The raw data supporting the conclusions of this article will be made available by the authors, without undue reservation.

## Author contributions

WS and JM conceived and designed the experiments. RJ performed the experiments and wrote the manuscript. JM and YW analyzed the data. JM contributed to the reagents, materials, and analysis tools. HZ revised the manuscript. All authors contributed to the article and approved the submitted version.
