# Supplementary material for: Development of a sensor-based site-specific N topdressing algorithm for a typical leafy vegetable
Source: Front Plant Sci. 2022 Aug 26;13:951181. doi: 10.3389/fpls.2022.951181 (PMC9479683; doi:10.3389/fpls.2022.951181)
Supplement: Supplementary file 1 [file Data_Sheet_1.docx]

Supplementary Material

**Supplementary Table 1** NDVI and RVI measurements at rosette stage and yield of bok choy as affected by N management and plant density in Year I, II and III.

| **Year** | **N management** | **NDVI** | | | **RVI** | | | **Yield (Mg ha^-1^)** | | |
| --- | --- | --- | --- | --- | --- | --- | --- | --- | --- | --- |
| Year I | N1 | 0.447 b | | | 2.617 b | | | 6.855 b | | |
|  | N2 | 0.488 a | | | 2.910 a | | | 9.043 a | | |
|  | N3 | 0.499 a | | | 2.997 a | | | 10.32 a | | |
|  | N4 | 0.496 a | | | 2.973 a | | | 10.31 a | | |
| Year II | N1 | 0.340 b | | | 2.035 b | | | 4.365 b | | |
|  | N2 | 0.382 ab | | | 2.235 ab | | | 4.794 b | | |
|  | N3 | 0.433 a | | | 2.545 a | | | 6.302 a | | |
|  | N4 | 0.408 a | | | 2.384 ab | | | 6.476 a | | |
| Year III | N0-HD | 0.359 b | | | 2.122 b | | | 4.500 cd | | |
|  | N1-HD | 0.348 b | | | 2.071 b | | | 4.884 c | | |
|  | N2-HD | 0.382 ab | | | 2.238 ab | | | 6.338 bc | | |
|  | N3-HD | 0.403 a | | | 2.349 a | | | 7.849 ab | | |
|  | N4-HD | 0.409 a | | | 2.388 a | | | 8.026 a | | |
|  | N0-LD | 0.276 c | | | 1.176 b | | | 3.150 c | | |
|  | N1-LD | 0.280 bc | | | 1.778 b | | | 3.585 c | | |
|  | N2-LD | 0.309 ab | | | 1.897 ab | | | 3.889 bc | | |
|  | N3-LD | 0.325 a | | | 1.966 a | | | 4.800 ab | | |
|  | N4-LD | 0.335 a | | | 2.010 a | | | 5.164 a | | |
|  | | Analysis of variance | | | | | | | | |
| Source of variation | | df | *F*-value | *P* | df | *F*-value | *P* | df | *F*-value | *P* |
| Year I | N management | 3 | 4.935* | 0.032 | 3 | 4.400* | 0.042 | 3 | 8.641* | 0.007 |
| Year II | N management | 3 | 4.171* | 0.047 | 3 | 3.663* | 0.063 | 3 | 16.25* | 0.001 |
| Year III | N management | 4 | 12.20** | 0.000 | 4 | 10.90** | 0.000 | 4 | 16.98** | 0.000 |
|  | density | 1 | 124.5** | 0.000 | 1 | 109.6** | 0.000 | 1 | 68.13** | 0.000 |
|  | N management*density | 4 | 0.140 | 0.965 | 4 | 0.239 | 0.913 | 4 | 1.935 | 0.144 |
| Pooled | N management | 4 | 14.65** | 0.000 | 4 | 1.133** | 0.000 | 4 | 29.17** | 0.000 |
|  | density | 1 | 81.14** | 0.000 | 1 | 31.21** | 0.000 | 1 | 66.32** | 0.000 |
|  | N management*density | 4 | 0.091 | 0.985 | 4 | 0.302 | 0.874 | 4 | 1.884 | 0.135 |

Note: *: means significant correlation at the *P* < 0.05 level, **: significant correlation at the *P* < 0.01 level.

**Supplementary Table 2** The correlation coefficients (R^2^) of between sensor-based measurements (NDVI and RVI) and yield potential without additional topdressing N application (YP_0_) of bok choy at different growth stages among years at different densities of high density (HD) and low density (LD) using three different equations.

| Growth  Stage | HD | | | | | |  | LD | | | | | |
| --- | --- | --- | --- | --- | --- | --- | --- | --- | --- | --- | --- | --- | --- |
|  | NDVI | | | RVI | | |  | NDVI | | | RVI | | |
|  | L^1^ | E^2^ | P^3^ | L | E | P |  | L | E | P | L | E | P |
| 5–6 True-Leaf | 0.69** | 0.69** | 0.69** | 0.68** | 0.68** | 0.68** |  | 0.71** | 0.71** | 0.70** | 0.72** | 0.72** | 0.72** |
| Rosette | 0.78** | 0.81** | 0.79** | 0.81** | 0.84** | 0.82** |  | 0.79** | 0.77** | 0.79** | 0.78** | 0.76** | 0.77** |
| Cupping | 0.16 | 0.16 | 0.16 | 0.16 | 0.15 | 0.16 |  | 0.03 | 0.03 | 0.03 | 0.03 | 0.03 | 0.03 |
| Mature | 0.01 | 0.01 | 0.01 | 0.01 | 0.01 | 0.01 |  | 0.20 | 0.20 | 0.20 | 0.20 | 0.21 | 0.20 |

Note: ^1^: L represented the linear equation; ^2^: E represented the exponential equation; ^3^: P represented the power equation, *: means significant correlation at the *P* < 0.05 level, **: significant correlation at the *P* < 0.01 level.

**Supplementary Table 3** N concentration of harvest bok choy from different fields in the research region.

| Field number | N concentration (%) |
| --- | --- |
| Field 1 | 5.26 |
| Field 2 | 5.24 |
| Field 3 | 5.35 |
| Field 4 | 5.64 |
| Field 5 | 5.47 |
| Field 6 | 5.62 |
| Field 7 | 5.57 |
| Field 8 | 5.93 |
| Field 9 | 5.82 |
| Field 10 | 5.12 |
| Field 11 | 5.46 |
| Field 12 | 5.26 |
| Field 13 | 5.24 |
| Field 14 | 5.35 |
| Field 15 | 5.64 |
| Field 16 | 5.47 |
| Field 17 | 5.62 |
| Field 18 | 5.57 |
| Field 19 | 5.93 |
| Field 20 | 5.82 |
| Field 21 | 5.12 |
| Field 22 | 5.47 |
| Field 23 | 5.53 |
| Field 24 | 5.46 |
| Average | 5.50 |

**Supplementary Table 4** Descriptive statistics of sensor-based measurements (NDVI and RVI) of bok choy at different growth stages across years at different densities of high density (HD) and low density (LD).

| Index | Growth Stages | HD | | |  | LD | | |  | HD & LD | | |
| --- | --- | --- | --- | --- | --- | --- | --- | --- | --- | --- | --- | --- |
|  |  | Mean^1^ | SD^2^ | CV^3^ |  | Mean | SD | CV |  | Mean | SD | CV |
| NDVI | 5–6 True-Leaf | 0.28 | 0.09 | 31.65 |  | 0.24 | 0.10 | 39.32 |  | 0.26 | 0.09 | 36.20 |
|  | Rosette | 0.43 | 0.06 | 13.60 |  | 0.34 | 0.06 | 16.18 |  | 0.38 | 0.07 | 18.42 |
|  | Cupping | 0.57 | 0.04 | 7.40 |  | 0.44 | 0.05 | 10.87 |  | 0.50 | 0.08 | 15.83 |
|  | Mature | 0.58 | 0.03 | 5.78 |  | 0.48 | 0.04 | 8.95 |  | 0.53 | 0.06 | 11.59 |
| RVI | 5–6 True-Leaf | 1.81 | 0.36 | 19.66 |  | 1.69 | 0.35 | 20.96 |  | 1.75 | 0.36 | 20.79 |
|  | Rosette | 2.52 | 0.36 | 14.34 |  | 2.07 | 0.28 | 13.48 |  | 2.29 | 0.40 | 17.32 |
|  | Cupping | 3.65 | 0.45 | 12.38 |  | 2.57 | 0.31 | 12.09 |  | 3.11 | 0.67 | 21.47 |
|  | Mature | 3.74 | 0.38 | 10.25 |  | 2.89 | 0.35 | 12.08 |  | 3.31 | 0.57 | 17.13 |

Note: ^1^: average value; ^2^: standard deviation of the mean; ^3^: coefficient of variation (%).
